# Early Phylogenetic Diversification of SARS-CoV-2: Determination of Variants and the Effect on Epidemiology, Immunology, and Diagnostics

**DOI:** 10.3390/jcm9061615

**Published:** 2020-05-26

**Authors:** Rene Kaden

**Affiliations:** Department of Medical Sciences, Uppsala University, 752 36 Uppsala, Sweden; rene.kaden@medsci.uu.se

**Keywords:** molecular epidemiology, SARS-CoV-2, COVID-19, virus variant detection

## Abstract

The phylogenetic clustering of 95 SARS-CoV-2 sequences from the first 3 months of the pandemic reveals insights into the early evolution of the virus and gives first indications of how the variants are globally distributed. Variants might become a challenge in terms of diagnostics, immunology, and effectiveness of drugs. All available whole genome sequence data from the NCBI database (March 16, 2020) were phylogenetically analyzed, and gene prediction as well as analysis of selected variants were performed. Antigenic regions and the secondary protein structure were predicted for selected variants. While some clusters are presenting the same variant with 100% identical bases, other SARS-CoV-2 lineages show a beginning diversification and phylogenetic clustering due to base substitutions and deletions in the genomes. First molecular epidemiological investigations are possible with the results by adding metadata as travelling history to the presented data. The advantage of variants in source tracing can be a challenge in terms of virulence, immune response, and immunological memory. Variants of viruses often show differences in virulence or antigenicity. This must also be considered in decisions like herd immunity. Diagnostic methods might not work if the variations or deletions are in target regions for the detection of the pathogen. One base substitution was detected in a primer binding site.

## 1. Introduction

The ongoing pandemic caused by the severe acute respiratory syndrome coronavirus 2 (SARS-CoV-2) resulted in 1,773,084 confirmed cases and 111,652 deaths worldwide as of April 13, 2020 [1].

As viruses, especially RNA viruses, usually have a high mutation rate [2], the fast evolution becomes visible, beside others, through fast adaption to new hosts or through variations in immunity response or in virulence [3]. Variants are phylogenetically clustered. Local SARS-CoV-2 clusters were identified by Pung et al. in 2020 in Singapore [4]. The aim of this study is the visualization of the early evolution within the first 3 month period after first detection of the SARS-CoV-2 in humans with a selection of 95 representative sequences from different sampling dates and from different countries. Selected variants were analyzed regarding the prediction of possible consequences for diagnostics and immune response or medication.

## 2. Experimental Section

All available SARS-CoV-2 sequences (accessed March 16, 2020) were downloaded from the NCBI database. Inclusion of metadata on the origin of the samples and the primary analysis were done in Geneious version 8.1.5. [5]. Only sequences in size intervals of 29,325 bases and 29,981 bases were further analyzed.

The remaining sequences from 13 countries (*n* = 93) and from the cruise ship Diamond Princess (*n* = 2) were annotated in Geneious based on the index case sequence [6] MN908947. A ClustalW alignment was performed including all 95 sequences. The phylogenetic tree was calculated in the Geneious FastTree plugin using a generalized time-reversible model and optimizing the Gamma20 likelihood. Genes of interest were separately aligned and translated into amino acid sequences, and the antigenic regions as well as the secondary protein structure were predicted with the EMBOSS 6.5.7 tool [7]. Primer sequences were copied from the corresponding publications [8,9,10] and re-evaluated in Geneious.

## 3. Results

### 3.1. Phylogeny and Early Clusters

**Figure 1:**
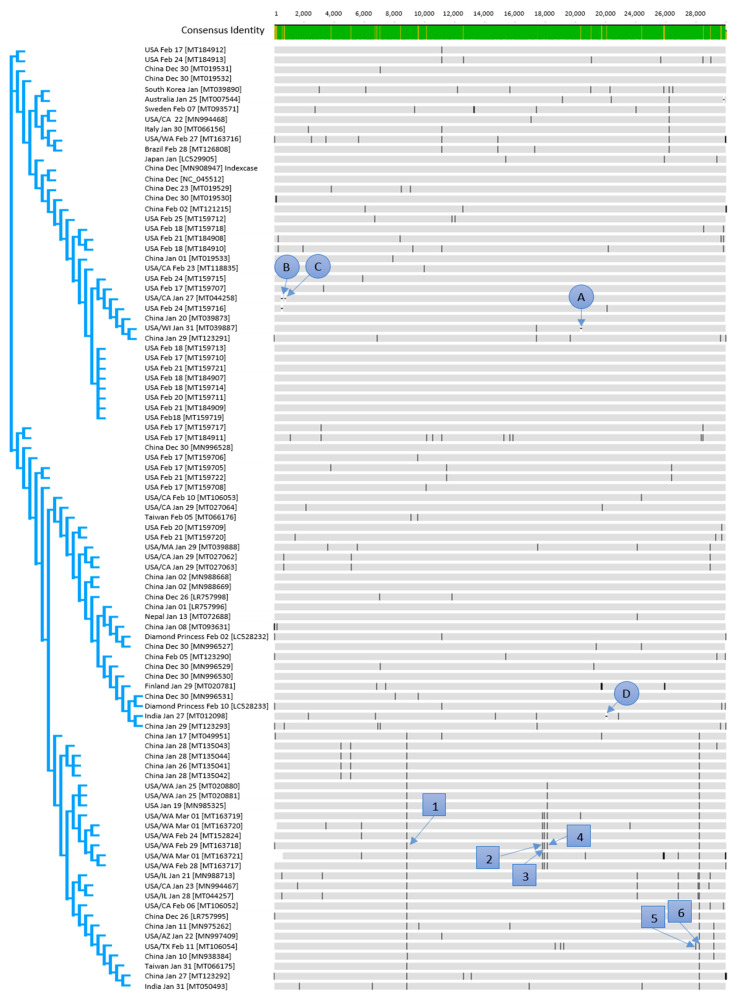
Alignment of all available sequences of SARS-CoV-2 from the NCBI database (March 16, 2020); numbers in squares are representing variants, letters in a circle are representing gaps.

All specified dates that are connected to the NCBI accession numbers are the sampling dates of patients. All countries are the countries in which the case was isolated, even if it was imported (Figure 1).

The nucleotide identity between all the available sequences was in the interval of 100% to 99.47%. The 100% identity was observed for the following samples (NCBI accession numbers): MN988668, MN988669 (both January 2, China), MT159710 (February 17, USA), MT159707, MT159713, MT159714, MT159719 (all February 18, USA), MT159711 (February 20, USA), MT159709, and MT159721 (both February 21, USA). The highest genetic divergence with 99.47% homolog bases was observed between the sequences MT163721 (March 1, USA/WA) and MT020781 (January 29, FIN). This high divergence was mainly caused by undetermined bases in the MT020781 sequence. The sequence was included in this study as it was the only available sequence from Finland. By excluding the sequences MT163721 and MT020781, all other sequences showed a maximal sequence divergence of about 0.19%, which corresponds to a maximum of 57 bases.

Three clusters established within the first days of the pandemic (Figure 1) as sequences from each of the three main clusters occurred already in December 2019.

### 3.2. Genetic Variants

The effect of genetic variants (Figure 1) on amino acid level was investigated on six selected, predominant variants numbered with 1–6 in Table 1. Furthermore, deletion of triplets (letters A–D in Figure 1) was observed. The gaps are representing 3 base- (A and D), 15 base- (B), and 9 base- (C) deletions. Due to the triplet deletions, no frameshift occurs.

**Figure 2:**
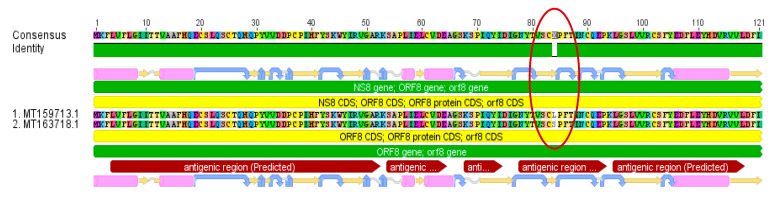
NS8 gene amino acid alignment (MT159713/MT163718) with a confirmed variation Leucine → Serine in an antigenic region occurring in 27% of all analyzed sequences.

While two of the investigated base substitutions resulted in silent mutations, the other four led to amino acid changes. Two of those four amino acid substitutions are located in predicted antigenic regions (Figure 2). A total of 27% of all examined sequences developed this Leucine → Serine variation in the NS8 gene in a predicted antigenic region.

### 3.3. Diagnostics

One aim of this study was the investigation of variants in primer binding sites (possible false negative results), not a full evaluation against close related species (false positive results). The primers that were developed by Chan et al. [8], Chu et al. [9], and Corman et al. [10] were re-evaluated in this study again after a re-evaluation of Wang et al. [11].

While the primer pair for the spike gene S, which was developed by Chan et al. [8] (Fw: CCT ACT AAA TTA AAT GAT CTC TGC TTT ACT; Rv: CAA GCT ATA ACG CAG CCT GTA), aligned 100%, the primers (Fw: **C**AA **G**TG GGG TAA GGC TAG ACT TT; Rv: A**C**T TAG GAT AAT CCC AAC CCA T) for the RNA-dependent RNA polymerase (RdRp) gene contains 2 mismatches, C/T and G/A in the forward primer and 1 C/T in the reverse primer (marked bold), for all 95 sequences in this study.

All primers and probes that were published by Chu et al. [9] aligned 100% to all sequences of this study (Fw: TGG GGY TTT ACR GGT AAC CT; Rv: AAC RCG CTT AAC AAA GCA CTC; Probe: TAG TTG TGA TGC WAT CAT GAC TAG) and (Fw: TAA TCA GAC AAG GAA CTG ATT A; Rv: CGA AGG TGT GAC TTC CAT G; Probe: GCA AAT TGT GCA ATT TGC GG).

Of the three primer sets which were developed by Corman et al. [10], the primer for the nucleoprotein gene (Fw: CAC ATT GGC ACC CGC AAT C; Rv: GAG GAA CGA GAA GAG GCT TG; Probe: ACT TCC TCA AGG AAC AAC ATT GCC A) matched 100% to the here described dataset. Probe 1 and the reverse primer for the ORF1b (Fw: GTG ARA TGG TCA TGT GTG GCG G; Probe 1: CCA GGT GGW ACR TCA TCM GG**T** GAT GC; Probe 2: CAG GTG GAA CCT CAT CAG GAG ATG C; Rv: CAR ATG TTA AA**S** ACA CTA TTA GCA TA) showed one mismatch, T/A and S/A (marked bold), respectively, throughout all 95 sequences. The primer set of the E gene aligned 100% with one exception in the probe for sequence (MT039890), which had a T/A variation (underlined) (Fw: ACA GGT ACG TTA ATA GTT AAT AGC GT; Rv: ATA TTG CAG CAG TAC GCA CAC A; Probe: ACA CTA GCC ATC CTT ACT GCG CTT CG).

## 4. Discussion

The genomes that were used in this study were generated by different sequencing methods. Salipante et al. [12] determined a technical error of the Illumina sequencing method including all steps from library preparation to bioinformatics of 0.467 ± 0.333 (*n* = 19). As the presented and here discussed variants occurred in different sequences in different institutes, the detection of variants is reliable.

The SARS-CoV-2 sequence starts clustering, and several stable clusters are already established. This effect was expected and was described for other RNA viruses before [2].

The sequences LC528232 and LC528233 (February 2 and February 10, respectively) are derived from patients from the cruise ship Diamond Princess, representing 2 variants with a total of 28 bases difference. Most differences were detected at the beginning and at the end of the sequence. This could also be explained by sequencing artefacts. However, if another sequencing confirms this finding, at least two passengers caused the outbreak on the Diamond Princess as the virus can hardly gather 28 variations within 8 days.

Variation 1 (Figure 1) occurred only a few days after the index case (MN908947) on the 23rd of December in 2019 (MT019529). As this mutation, which resulted in a silent mutation, has no significance on the virus evolution in terms of virulence or resistance, the 2 established variants might be probably stable, and the C/T variation can be used for epidemiological investigations. This can also be expected for variation 4 (silent mutation) which was detected in the sequence (MN985325) derived from a US strain on 19 January 2020. The base substitutions 2 and 3 with an unknown effect occurred also for the first time in strains from the USA on 24 February in 2020. Variant 6, which occurred first in the sequence LR757995 on 26 December in 2019 and is from China in a predicted antigenic region, is present throughout March, mainly in sequences from the USA in the present study. The observed base substitutions or base deletions which led to amino acid substitutions/deletions might result in different immune reactions of the different genetic variants [13]. Such effects could be problematic in terms of the expected immunity against SARS-CoV-2.

As variations can also have an impact on virulence, maybe less virulent variants can be identified with the help of the variant determination. It would be an advantage in health care if most virulent strains, once detected and described, can be detected very fast.

A challenge in diagnostics, caused by variants, is base substitution and deletion in primer binding sites [14]. False negatives occur as a result of such events. Regarding the actual situation, an undetected spread of SARS-CoV-2 would have a dramatic impact. 

The primer sequences published by Chan et al. [8] had 2 mismatches in the forward primer and one in the reverse primer of the RdRp gene in all 95 sequences of this study. This is similar to the results of the study of Wang et al. [11] who analysed these primers with a dataset of 95 sequences as well.

Of the 3 primer sets which were published by Corman et al. [10], the combination with the target on the nucleoprotein gene gave 100% homology while the ORF1b target gene primer set showed one T/A mismatch in probe 1, which was reported by Wang et al. [11] before and another S/A mismatch in the reverse primer, based on the sequences of all 95 sequences of this study. One variant was detected within the whole dataset which had a variation in the primer binding site of the probe [10] of the E gene.

In contrast to the study of Wang et al. [11], the primer sets which were developed by Chu et al. [9] gave a 100% match to all sequences in this study. Wang et al. [11] detected 3 mismatches in a probe for the nucleoprotein gene, which could not be confirmed with the dataset of the here presented study.

The discrepancies in the published primer sequences might be caused by the fact that the authors had only a limited number of sequences available for the primer design at the beginning of the pandemic. Thus, the primers must be re-evaluated in certain time intervals including especially the latest sequences. Furthermore, new uploaded sequences should be examined regularly for variations in primer binding sites as it was shown in this study that one probe was constructed in a region in which already the first variation occurred.

The overall aim of the study was the comparison of a selection of SARS-CoV-2 sequences from different countries and time points within the first 3 months of the pandemic to show the early evolution of this virus. Thus, only a selection of 95 full-length sequences from the NCBI database was chosen. Additional variants to the here presented 95 strains are expected to be present all over the world. A limitation of the study is the lack of metadata information like the travelling history of the patients. These data were only available for sequence MT126808 (February 28, Brazil) from a patient who travelled to Italy and Switzerland. However, the data are available in the institutes that submitted the sequences to the database and can be combined with this study for source tracing and risk assessments.

## Figures and Tables

**Table 1:** Selection of 6 regions with base substitutions; variants are coded as initial base/amino acid-position after startcodon-base substitution; reference sequence for all analyses: MT159713.

**Selected Region**	1	2	3	4	5	6
**Variant Sequence**	MT163718	MT163718	MT163718	MT163718	LR757995	MT163718
**Total number sequences with variation**	26	6	6	9	2	26
**Gene**	orf 1 ab gene, nsp4 peptide	orf 1 ab gene, nsp13 peptide	orf 1 ab gene, nsp13 peptide	orf 1 ab gene, nsp14 peptide	NS8 gene	NS8 gene
**Function**	contains transmembrane domain	helicase and others	helicase and others	exonuclease	unknown	unknown
**Variant Nucleotide**	C8517T	C17483T	A17593G	C17795T	C32T	T251C
**Variant Amino Acid**	S2839S	P5828L	Y5865C	L5932L	T11I	L84S
**Consequence**	silent mutation	effective variant	effective variant	silent mutation	effective variant	effective variant
**Substitution in antigenic region**	not relevant	no	no	not relevant	yes	yes
